# Assessment of the Effects of Zoledronic Acid Therapy on Bone Metabolic Indicators in Hormone-Resistant Prostate Cancer Patients with Bone Metastatasis

**DOI:** 10.5402/2011/392014

**Published:** 2011-05-04

**Authors:** Abdullah Demirtas, Nurettin Sahin, Mehmet Caniklioglu, Mustafa Kula, Oguz Ekmekcioglu, Atila Tatlisen

**Affiliations:** ^1^Department of Urology, Erciyes University Faculty of Medicine, Kayseri, Turkey; ^2^Department of Nuclear Medicine, Erciyes University Faculty of Medicine, Kayseri, Turkey

## Abstract

*Purpose*. Assessment of effects of zoledronic acid therapy on bone metabolic indicators in hormone-resistant prostate cancer patients with bone metastasis. *Material and Methods*. Hormone-resistant prostate cancer patients who were identified to have metastases in their bone scintigraphy were taken to trial group. Before administration of zoledronic acid, routine tests for serum calcium, total alkalen phosphates were studied. Sample sera for bone metabolic indicators BALP, PINP, and ICTP were collected. Bone pain was assessed via visual analogue scale and performance via Karnofsky performance scale. Four mg zoledronic acid was administered intravenously once a month. *Results*. When serum levels of bone forming indicators PINP; BALP were compared before and after therapy, there were insignificant decreases (*P* = .33, *P* = .21, resp.). Serum levels of bone destruction indicator ICTP was compared, and there was a significant decrease after zoledronic acid therapy (*P* = .04). When performances of the patients were compared during therapy period, performances decreased significantly due to progress of illness (*P* = .01). All patients had ostalgia caused by bone metastases at various degrees. Significant decrease in pain scores was observed (*P* < .01). *Conclusion*. Zoledronic acid therapy decreased bone destruction and was effective in palliation of pain in patient with bone metastasis. Using bone metabolic indicators during followup of zoledronic acid therapy might be useful.

## 1. Introduction

Prostate cancer is the fourth most common male malignancy in the world and the first in North America [1]. Treatment for the illness should be planned in accordance with the stage, age, life expectance, and additional illnesses. The first therapy in patients with acute stage prostate cancer is to decrease androgens via medical or surgical castration. However, in most of the cases the illness progresses throughout this therapy. Average life span of the hormone-resistant prostate cancer patients is about eighteen months and no contribution to the life span through extra therapies could be demonstrated [2]. 

In prostate cancer potential of bone metastases is high. Bone lesions seen in prostate cancer are of osteoblastic characteristic, and they make extreme bone reconstruction and destruction around metastatic areas [3]. Therefore local osteopenia and widespread bone metabolism disorders are seen in patients with advanced stage prostate cancer.

Traditional approach in the treatment of bone metastatic patients is the application of supportive or palliative therapies besides standard anticancer therapy [4]. Serious ostalgia may be cured with radiotherapy or radio-nuclides and many patients also take systemic analgesic therapy via nonsteroidal anti-inflammatory medicines or opioids. 

Recently, bisphosphonate therapy has become highly efficient alternative in preventing secondary skeletal complications in bone metastases [4]. Bisphosphonates both join in active bone metabolism and especially adhere to bone. During bone degeneration they are released from bone matrix and they potentially decrease osteoclast activity and life span. Thus bone degeneration decreases [5]. Bisphosphonates are used to decrease skeletal complications, palliate pain and prevent metastatic bone disorders [6]. 

We aimed at assessment of effects of zoledronic acid therapy on bone metabolic indicators in hormone-resistant prostate cancer patients with bone metastasis.

## 2. Material and Methods

Patients receiving therapy were identified to have metastases in their bone scintigraphy and resistant to hormone therapy were taken to the trial group regardless of the number of metastases. Before the initiation of zoledronic acid therapy, routine tests prostate-specific antigen (PSA), serum calcium (Ca), and total alkaline phosphates (TALP) were studied. The sample sera for bone metabolic indicators that bone-specific alkaline phosphates (BALP), aminoterminal propeptide of type I procollagen (PINP), carboxyterminal pyridinoline cross-linked telopeptide of type I collagen (ICTP) were kept at −70°C.

According to the individual positions of the patients radiotherapy for vertebral metastases, nonsteroid anti-inflammatory medicines and opioids for pain palliation were additionally given. 

The degree of bone pain was assessed via visual analogue scale (VAS) and their performances via Karnofsky performance scale. All patients were evaluated with dual energy X-ray absorptiometry (DEXA) test before the start of therapy.

 Four mg zoledronic acid was administered intravenously to each patient once a month in fifteen minutes. Before each administration serum creatinine and calcium levels were determined routinely. All patients were given an additional daily 500–1500 mg oral dose calcium and 300–900 IU vitamin D during therapy.

During followup PSA, serum calcium, serum total alkaline phosphates test were routinely studied every three months. The sera for bone metabolic indicators (BALP, PINP, and ICTP) were kept at −70°C.

Patients did not have other bone disorders (osteomyelitis, Paget's disease, etc.) that might affect serum levels of bone metabolic indicators.

Levels of bone metabolic indicators before and after therapy were determined. 

Carboxyterminal pyridinoline cross-linked telopeptide of type I collagen (ICTP) and aminoterminal propeptide of type I procollagen (PINP) tests were studied using RIA method (Orion Diagnostica, Espoo Finland). Reference interval for ICTP and PINP was 2.1–5.0 mg/L, 19–102 *μ*g/L, respectively. Bone alkaline phosphates tests were studied through RIA method (Tandem-R Ostase Beckman Coulter), and reference interval was 5.50–17.50 *μ*g/L.

Statistical analyses were performed in SPSS 15 program with Wilcoxon Signed Ranks Test. *P* < .05 was accepted as statistically significant.

## 3. Result

Fifteen patients with hormone-resistant prostate cancer and with bone metastases in their bone scintigraphy, whose ages ranging 58–85 (median 73) were evaluated. Duration of the followup was 2–15 months (median four months). External radiotherapy for spinal cord compression symptoms developed due to vertebral metastases was applied to eight patients (3000 cGy).

Control tests were performed in the second month for three patients and in the third month to others. Twelve patients have been orchidectomised and three were under androgen blockage (receptor blockage and LHRH agonists).

In the followup period, in ten patients PSA values were 100 ng/ml and in the other five patients, PSA values were tending to increase. But this increase did not reach statistical significance.

In DEXA tests applied before the therapy all patients were found to have decreased bone mineral density in lumbar vertebrae and femur cervix (*T* score < −2.5, osteoporosis). Although the zoledronic acid therapy patients were given a daily 500–1500 mg oral dose of calcium and 300–900 IU vitamin D significant decrease in serum calcium levels after the therapy was observed (Table 1).

When the patients TALP levels were compared, there were statistically significant decreases after therapy (*P* < .01) (Table 1).

Although there was a decrease in other bone construction indicator BALP, this decrease (median before and after therapy 29.40 *μ*g/L, resp.) was not statistically significant (*P* = .21) (Table 1).

When serum levels of bone destruction indicator ICTP were compared, significant decrease after zoledronic acid therapy was determined (median before and after therapy 7.76 *μ*g/L and 5.65 *μ*g/L, resp.) (Table 1).

When performances of the patients were compared a statistically significant decrease in performances of the patients due to the progression of the illness during the therapy (*P* = .01) (Table 2) was observed. Six patients died due to progression of the diseases during followup period. All patients had bone pain in different degrees assessed via VAS caused by bone metastases. When pain before and after therapy was assessed, significant decreases in pain scores were determined (*P* = .01) (Table 2). Due to this fact patients need for analgesics decreased. 

In this study zoledronic acid was tolerated well. There were no patients giving up therapy because of adverse effects. Adverse effects were mild to moderate in all of the patients followed by the initial administration but the symptoms disappeared within 24 hours without any need for additional therapy. Adverse effects were nausea, myalgia, mild tremor in hands, and fever not higher than 38°C. 

## 4. Comment

Although bone lesions seen in prostate cancer are of osteoblastic characteristic, they make extreme bone construction and destruction around metastatic areas [3]. Thus both bone construction and destruction indicator levels in serum become high.

BALP is localized in the plasma membrane of osteoblasts and is released into circulation during bone mineralization process [7]. It is an indicator of matrix maturation phase [8]. BALP gives more specific information about bone lesions than serum TALP. PINP is another bone construction indicator. It shows constructions of collagen. It is indicator of early or proliferation phase. ICTP is biochemical indicator of bone destruction. It is product of collagen destruction [7].

Androgen blockage have been a basic method in advanced prostate cancer for a long time, but it causes a continuing decrease in bone mineral density during therapy. 183 patients receiving hormone therapy for advanced prostate cancer was studied by Weston et al. bone mineral density were measured at the 12th and 24th months of the therapy. In the 12th month 36% of the patients and in the 24th month 62% of the patients had osteoporosis [9]. Risk of bone fracture after androgen blockage therapy over five years increased dramatically. This risk reaches around 50% after a 9-year therapy [10].

Besides old age's and androgen blockage therapy's being known risk factors for osteoporosis, prostate cancer is also a cause for osteoporosis alone. Hussain and colleagues [11] assessed 174 patients having newly diagnosed advanced prostate cancer. Patients were evaluated with DEXA test before any kind of therapy for prostate cancer. Results were compared to a control group of 106 patients consisting of people of similar ages not having prostate cancer but having different urological and medical illnesses. In patients having advanced untreated prostate cancer there was a prominent superabundance in osteoporosis in comparison to the patients in the control group (42% in patients with prostate cancer and 27% in the control group, *P* = .02). Although age, smoking, and family background of osteoporosis increase the risk of osteoporosis, advanced prostate cancer is an independent cause for osteoporosis alone. These investigators suggest that bone mineral density be measured routinely before androgen blockage therapy is started in patients with advanced prostate cancer [11]. 

In another clinical study including 15 osteoporotic patients in whom radical cystectomy had been performed, both construction and destruction metabolic bone markers increased significantly following zoledronic acid treatment (unpublished data).

Today palliative therapy for patients having malign bone disorders consist of radiotherapy, chemotherapy, hormone therapy, surgery, and intravenous bisphosphonates. Randomized placebo controlled trials have indicated that intravenous bisphosphonates decreases the frequency of skeletal complications with malign bone disorders in patients with prostate cancer [12, 13].

Bone scintigraphy is frequently used in determining the site and degree of bone metastases. However the use of bone scintigraphy in the observation of the efficiency of the therapy is limited. It is costly and time-consuming process. It does not reflect the rapid skeletal response. Due to these negotiations, in the observation and efficiency determination of the therapy, early skeletal response might be assessed via biochemical bone metabolic indicators [14]. In a prospective trial carried out by Schoenberger and colleagues [15], bone metastases were found in 21 of 88 patients with malign tumors. In these patients bone scintigraphy was compared to bone metabolic indicators. Sensitivity of bone scintigraphy was 90%. The sensitivity and specificity of ICTP were 71%, and 42%, and PINP 24% and 96%, respectively. Although, bone scintigraphy is more expensive and time consuming it is still the most sensitive technique especially in terms of determining the site bone lesions and the frequency of bone disorder [15]. 

In a trial carried out among postmenopausal women it is determined that serum levels of bone construction and destruction indicators caused by osteoporosis were high [16]. 

Similarly in this trial it was found that both bone construction (TALP, BALP) and destruction (ICTP) indicators for patients being under observation for 2–15 months (median 4 month) were high. It was determined that there was a statistically significant decrease in serum levels of ICTP after zoledronic acid therapy (*P* < .05). 

In this study numbers of the patients were low and the observation period was short. Determining a significant decrease in serum levels of ICTP brought about the idea that serial measurements of ICTP levels may be an important guide in observation of zoledronic acid therapy; it is even determined in early controls and it was preserved in late controls and even decreased to normal serum levels in patients being under the longest observation of 15 months. 

Although there were decreases in serum levels of bone construction indicators (BALP, PINP) they were not statistically significant; these decreases in bone construction indicators might be an indicator of decrease in abnormal bone construction. Still bone construction indicators decreasing less in proportion to the bone destruction indicators may be due to respectively slowed down reactionary new bone construction continuing with the inhibition of osteoclasts by zoledronic acid. The decrease in bone construction indicators when compared to the initial levels although they were less affected than destruction indicators, and the significant decrease in serum calcium levels may be an indirect indicator of a better mineralization in the newly constructed bone. In a study it was reported that zoledronic acid therapy resulted in a 7.8% of increase in bone mineral density [17].

Bone metastases are the most common reason for the pain related to bones and sometimes require palliative radiotherapy [18]. It was reported that bisphosphonates have an analgesic effect on the pain in the bone in patients with bone metastases [19].

In this trial eight patients received radiotherapy due to ostalgia caused by vertebral involvement and spinal cord compression symptoms. At the same time zoledronic acid therapy was continued to be applied. Although performances of the patients decreased during observation due to progression in the illness there were significant decreases in pain scales. Additionally there was a decrease in patients' needs for analgesics. There is limited information about the optimal application period of zoledronic acid therapy in the literature. Bone disorders caused by prostate cancer are a serious life quality decreasing reason of morbidity. Still, there is not a primary efficient cure for bone disorders caused by prostate cancer. Newly developed, intravenously administered bisphosphonates are used with an increasing rate in the palliative therapy of bone disorders. Location and prevalence of the bone disorder and its response to the therapy are assessed by techniques like bone scintigraphy and DEXA which are expensive, time consuming, and not good reflectors of early therapy response. Due to difficulties in these techniques, serial measurement of serum levels of bone metabolic indicators which can assess the diagnosis and observation of the bone disorders caused by prostate cancer and especially the early response to the therapy may be useful. Therefore there is still a need for randomized trials in larger series with patients of longer observation period. Moreover it would increase the benefit expected, to develop more sensitive more specific bone indicators. 

## 5. Conclusion

Zoledronic acid therapy decreased bone destruction and was effective in palliation of the pain in patient with bone metastasis. Using bone metabolic indicators during the followup of zoledronic acid therapy might be useful.

## Figures and Tables

**Table 1:** Comparison of bone metabolic indicators and serum calcium levels in fifteen patients before and after zoledronic acid therapy.

	Before therapy median ± SE (min-max)	After therapy median ± SE (min-max)	*P*
TALP	600.00 ± 167.48	294.00 ± 110.00	.01
IU/L	(112.00–2390.00)	(108.00–1422.00)	

PINP	39.56 ± 84.29	35.73 ± 73.28	.33
*μ*g/L	(14.58–1000.00)	(6–1000.00)	

ICTP	7.76 ± 7.44	5.65 ± 5.13	.04
*μ*g/L	(2.89–93.11)	(2.00–63.80)	

BALP	29.40 ± 26.20	20.18 ± 38.10	.21
*μ*G/L	(7.63–319.73)	(3.92–480)	

Serum calcium	9.70 ± 0.15	9.0 ± 0.13	<.01
mg/dL	(8.70–10.60)	(8.20–9.80)	

TALP: Total alkline phosphates, PINP: amino-terminal propeptide of type I procollagen, ICTP: Carboxyterminal pyridinoline telopeptide of type I collagen, BALP: bone specific alkaline phosphates.

**Table 2:** Comparison of pain scores of fifteen patients before and after therapy.

	Before therapy median ± SE (min-max)	After therapy median ± SE (min-max)	*P*
Visual analogue	5.00 ± 0.41	1.00 ± 0.52	<.01
scale (VAS)	(3.00–8.00)	(0.00–6.00)	
